# Chloride Permeability of Damaged High-Performance Fiber-Reinforced Cement Composite by Repeated Compressive Loads

**DOI:** 10.3390/ma7085802

**Published:** 2014-08-11

**Authors:** Byung Jae Lee, Jung Hwan Hyun, Yun Yong Kim, Kyung Joon Shin

**Affiliations:** 1 R&D Center Manager, JNT INC, Korea; E-Mail: lbjae80@hanmail.net; 2 Department of Civil Engineering, Chungnam National University, 99 Daehak-ro, Yuseong-gu, Daejeon 305-764, Korea; E-Mails: jhhyun@cnu.ac.kr (J.H.H.); yunkim@cnu.ac.kr (Y.Y.K.)

**Keywords:** chloride permeability, high-performance fiber-reinforced cement composite(HPFRCC), compressive damage

## Abstract

The development of cracking in concrete structures leads to significant permeability and to durability problems as a result. Approaches to controlling crack development and crack width in concrete structures have been widely debated. Recently, it was recognized that a high-performance fiber-reinforced cement composite (HPFRCC) provides a possible solution to this inherent problem of cracking by smearing one or several dominant cracks into many distributed microcracks under tensile loading conditions. However, the chloride permeability of HPFRCC under compressive loading conditions is not yet fully understood. Therefore, the goal of the present study is to explore the chloride diffusion characteristics of HPFRCC damaged by compressive loads. The chloride diffusivity of HPFRCC is measured after being subjected to various repeated loads. The results show that the residual axial strain, lateral strain and specific crack area of HPFRCC specimens increase with an increase in the damage induced by repeated loads. However, the chloride diffusion coefficient increases only up to 1.5-times, whereas the specific crack area increases up to 3-times with an increase in damage. Although HPFRCC shows smeared distributed cracks in tensile loads, a significant reduction in the diffusion coefficient of HPFRCC is not obtained compared to plain concrete when the cyclic compressive load is applied below 85% of the strength.

## 1. Introduction

Over the past several decades, a massive number of concrete structures have been constructed in many countries. However, these structures have suffered from safety and serviceability problems due to the deterioration of concrete. For this reason, concrete durability has received a great amount of attention. The penetration of water, chloride and other aggressive ions into concrete is the most important factor in the deterioration of concrete [1]. Of these ions, the chloride ion poses the most significant durability problems, because it causes the corrosion of the steel reinforcement embedded in concrete. Particularly for concrete structures in marine environments, chloride-induced corrosion of steel reinforcement causes the spalling of concrete covers and the loss of steel cross-sections. As a result, the service life of such concrete structures decreases due to a lack of safety and serviceability [2].

It is well known that fiber reinforcements can reduce the width of cracks or can increase the toughness after cracking by bridging the cracks. However, because the reinforced fiber mainly comes into action after the cracking, the reduction of crack widths by using conventional fiber-reinforced concrete (FRC) is limited [3].

High-performance fiber-reinforced cement composite (HPFRCC) provides a well-controlled crack width compared to ordinary FRC. HPFRCC shows multiple cracking and strain hardening behaviors under tension. The key advantage of HPFRCC is its capability of reducing crack width under applied loads [4]. Engineered cementitious composite (ECC) typically has a tensile strain capacity of more than 2%, with spacing between multiple cracks at a saturation of less than 3 mm and maximum crack width of less than 100 μm. Microstructure optimization allows ECC to be made with a fiber content that is less than 2% by volume [5,6,7].

Because of this superior tensile strain hardening response, many studies have exploited the attractive behavior of HPFRCC, but have been mainly concerned with mechanical behaviors, such as ductility, deformation and strength. Only a limited number of researchers have studied the durability behavior of HPFRCC [8,9].

The development of cracking in concrete structures is one of the major factors influencing the durability of structures. Cracks accelerate the penetration of water and the diffusion of harmful ions, such as chloride, leading to damage and durability problems [10]. Therefore, the use of HPFRCC can contribute to enhancing the durability of structures by smearing one or several dominant cracks into many finely distributed cracks, even under severely loaded conditions.

Sahmaran *et al.* [11] proved that the effective diffusion coefficient of ECC was significantly lower than that of the reinforced mortar when the flexural deformation was applied. Lepech and Li [12] reported that the water permeability of cracked ECC and mortar is closely related with crack width by investigating cut flexural specimens. Charron *et al.* [13] showed that after subjecting it to stress, the permeability of ultra-high performance concrete (UHPC) was significantly reduced compared to that of plain concrete, since individual cracks were much finer in the UHPC than in the normal concrete.

However, most of the chloride tests on cracks have been conducted using cut samples from a flexural beam or tensile loaded specimens. In reality, since reinforced concrete structures resist compressive and tensile loads together, crack and damage patterns vary based on the types of loads. Therefore, permeability behavior needs to be investigated under variable loading conditions, including compressive loads, as well as tensile loads, and particularly under damaged conditions.

For plain concrete or ordinary FRC, several studies have been conducted in order to induce compressive damage and to measure transportation properties. In order to vary the damage induced, several levels of load (as a percentage of maximum stress) or various types of loading history (static or cyclic loads) have been adopted [14,15,16,17]. Banthia and Bhargava [14] measured the water permeability of plain and FRC with and without a static compressive stress applied and explored the effect of fiber and load level on the permeability. Saito and Ishimori [15] applied compressive static and repeated loads to the specimens and found that the chloride permeability of concrete increased at an increasing rate with its residual strain. Djerbi *et al.* [16,17] investigated the influence of transverse cracks and compressive loading on chloride diffusion with ordinary or high strength concrete, obtaining relations between the diffusion coefficient through the crack and crack width or residual strain and also found that this coefficient was not dependent on material parameters. However, it is not easy for researchers to find a related study for the HPFRCC.

Thus, the objective of this study is to explore the mechanical properties of HPFRCC under static and repeated compressive loading conditions and the chloride permeability of damaged specimens due to the repeated loads. In order to vary the level of damage, the maximum applied stress and number of load cycles are adapted as experimental parameters.

## 2. Experimental Method

### 2.1. Experimental Plan

**Figure 1:**
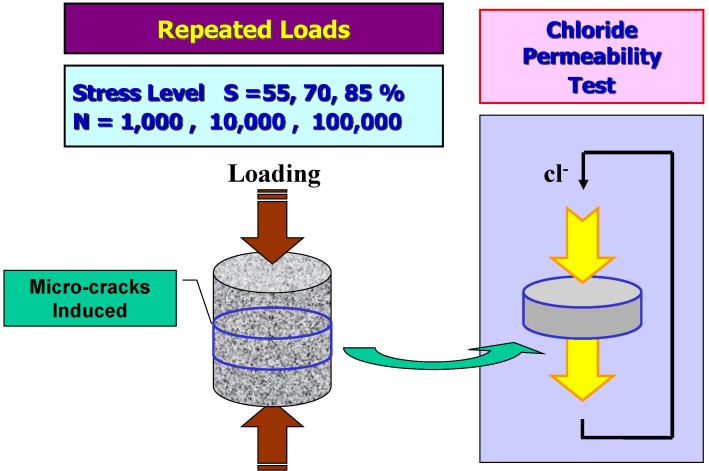
Schematic diagram of this experiment.

Several series of tests are planned for the investigation of the mechanical and durability performances of HPFRCC. Basic mechanical test are conducted in order to know the mechanical properties and behavior of HPFRCC. Compressive behaviors are tested with cylindrical specimens to measure the elastic modulus and compressive strength. A flexural test with beams is performed to investigate the tensile and cracking characteristics. After static tests are completed, various repeated compressive loads are applied to cylindrical specimens in order to induce internal damage. These damaged specimens are sliced and used for the chloride diffusion tests. Figure 1 shows the schematic diagram of this experiment.

### 2.2. Materials and Mixture Design

One typical HPFRCC mix is selected for the investigations. Ordinary Portland cement is used in the mixture, and a small amount of super plasticizer is added. The mix proportions used are a water-cement ratio (W/C) = 0.46 and sand-cement ratio (S/C) = 1.0. Polyvinyl alcohol (PVA) fibers with a 12-mm length and a 0.04-mm diameter are mixed together with a 2% volume fraction. Silica sand with an average diameter of 0.125 mm and a specific gravity of 2.65 is adopted as a fine aggregate. The use of silica sand with a smaller diameter than plain sand is known to reduce the fracture toughness of the matrix [18], and this low fracture toughness is one of the important necessary conditions needed to induce multiple cracks [19]. As a reference, specimens of plain concrete with a compressive strength of 28 MPa were also made.

### 2.3. Compressive Test

A cylindrical specimen with a 100-mm diameter and 200-mm height is cast and used to measure the compressive strength, elastic modulus and Poisson’s ratio. Each cylinder is ground before the tests. The specimens are cured for four months. The load is applied through a displacement control method, and complete load-deformation data are measured. Axial and lateral deformations are measured using displacement transducers and strain gauges, respectively.

### 2.4. Flexural Test

**Figure 2:**
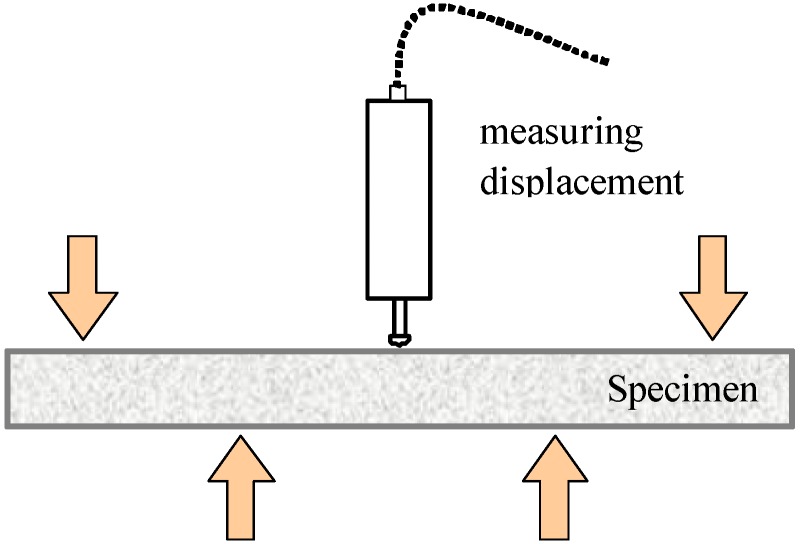
Four-point flexural test for mortar beams.

Flexural tests with beam specimens have been performed in order to investigate the tensile behavior, including cracking characteristics. A beam has a 100-mm width, 400-mm length and 30-mm height. The specimens are cured for four months. The load is applied through a displacement control method, and the complete load-deflection curve is measured. The deflection of the center is measured using displacement transducers of a strain gauge type. Figure 2 shows the test setup for this flexural test.

### 2.5. Specimens Damaged by Repeated Loads

In order to induce internal damage on the specimens, repeated compressive loads are applied. After four months of curing, the cylindrical specimens are loaded in compression to 55%, 70% and 85% of the static strength. 1000 cycles are applied for the 55% specimen, since the specimens failed before 10,000 cycles for this load level. 1000, 10,000 and 100,000 cycles are applied for specimens of 70% and 80% load levels. The ratio of the minimum load and the maximum loads is set to 0.2. The frequency of the loads is 2 Hz.

### 2.6. Damage Evaluation

When the cyclic loads are applied to the concrete specimens, damage is supposed to be accumulated, and microcracks form inside of the specimens. In order to evaluate the amount of damage, *i.e.*, microcracks of specimens, the residual axial and lateral strains are adopted as indirect measures. In addition, the specific crack area proposed by Loo [20] is adopted. The formula is derived on the assumption that the change in the cross-sectional area of a prismatic concrete specimen under uniaxial compression can be resolved into two parts: the elastic change in cross-sectional area due to Poisson’s ratio effects and the dilation due to microcracks. Therefore, the specific crack area can be calculated as follows:

ε_sca_ = 2(ε_lt_ − *ν*ε_ax_) (1)

where ε_sca_ is the specific crack area; ε_ax_ and ε_lt_ are the axial and lateral strain of concrete; and *v* is Poisson’s ratio.

### 2.7. Chloride Permeability Tests

**Figure 3:**
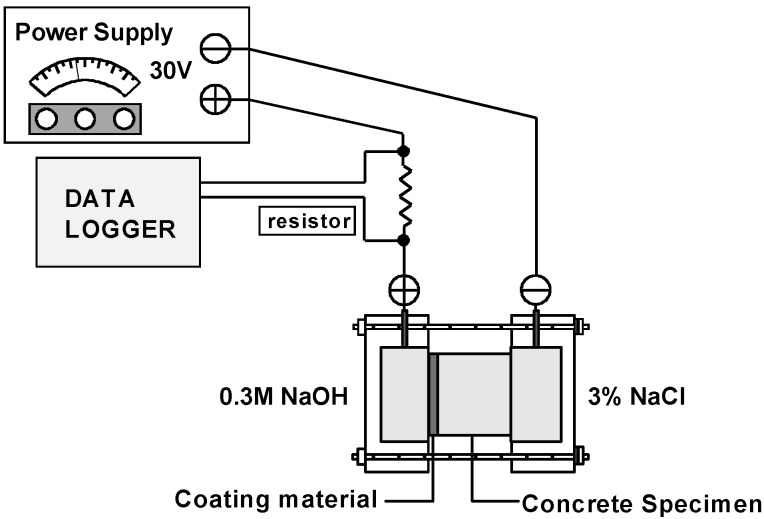
Test setup of chloride ion diffusion coefficients.

After the repeated loads are applied to the specimens, as explained in Section 2.5, the specimens are cut off from the central portions of the cylindrical specimens at a length of 50 mm, and chloride diffusion coefficients are measured. In this study, the method initially proposed by Luping and Nilsson [21] is adapted to evaluate the chloride permeability of damaged HPFRCC specimens, because the resistance to chloride penetration is one of the simplest measures to determine the durability of concrete. Figure 3 shows the test setup for this. The test is a non-steady-state migration test.

In the method, the chloride migration coefficient can be calculated from a penetration depth using Equation (2):

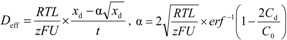
(2)
where *D*_eff_ is the effective diffusion coefficient (m^2^/s); *U* is the absolute value of the applied voltage (V); *T* is the average value of the initial and final temperatures in the anolyte solution (K); *L* is the thickness of the specimen (m); *x*_d_ is the average value of the penetration depths (m); and *t* is the test duration (h).

In order to measure the penetration depth, an external electrical potential is applied axially across the specimen and forces the chloride ions outside to migrate into the specimen. After a certain test duration, the specimen is axially split, and a silver nitrate solution is sprayed onto one of the freshly split sections. The chloride penetration depth can then be measured from the visible white silver chloride precipitation.

## 3. Results and Discussion

### 3.1. Static Behaviors

**Figure 4:**
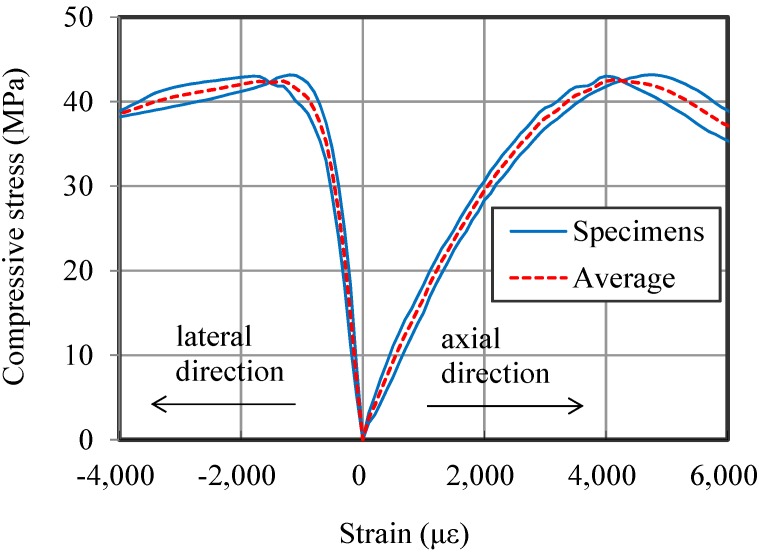
Compressive axial and lateral behavior of high-performance fiber-reinforced cement composite (HPFRCC).

Two cylindrical specimens are tested to measure the compressive behavior of HPFRCC. The result indicates that the average compressive strength of HPFRCC is 42.6 MPa. The stress-strain relationship is shown in Figure 4. The elastic modulus is calculated as 17 GPa using a secant modulus method. Poisson’s ratio is 0.19. These results show typical characteristics of ductile HPFRCC, such as a wide softening region after peak load, a low elastic modulus and a larger Poisson’s ratio than plain concrete [5,19].

**Figure 5:**
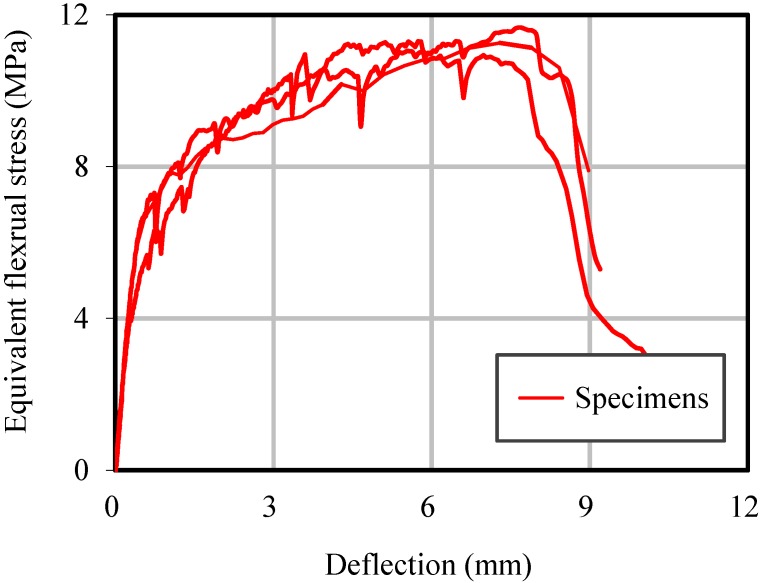
Flexural deformation of HPFRCC.

Flexural mechanical behaviors are also tested. The load-deflection relations are measured and shown in Figure 5. It can be seen that the tested specimens show fairly good ductility and strain hardening behavior, as observed in typical ductile HPFRCC.

**Figure 6:**
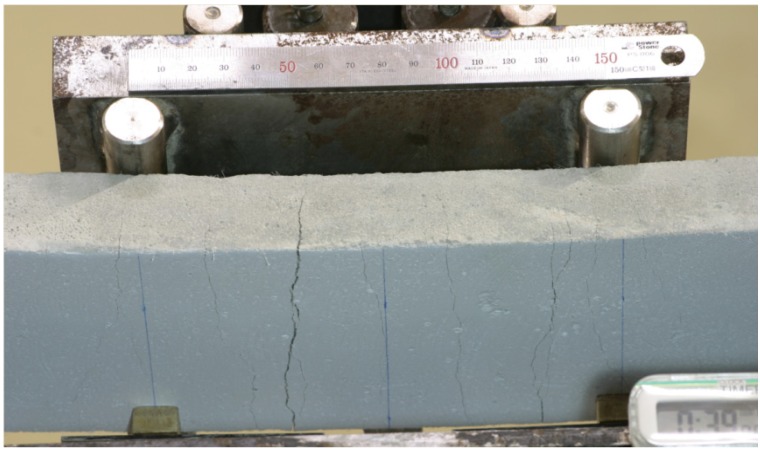
Typical crack patterns of the HPFRCC beam.

The crack patterns of the specimens after testing are shown in Figure 6. The average number of cracks in the tensile region is 7.07.

Static test results indicate that the mixture used in this study shows the unique behaviors of ductile HPFRCC or ECC. The specimens sustained the load continuously, even after peak load, and strain hardening behavior was observed with finely distributed microcracks in flexural tests.

### 3.2. Cyclic Behaviors

**Figure 7:**
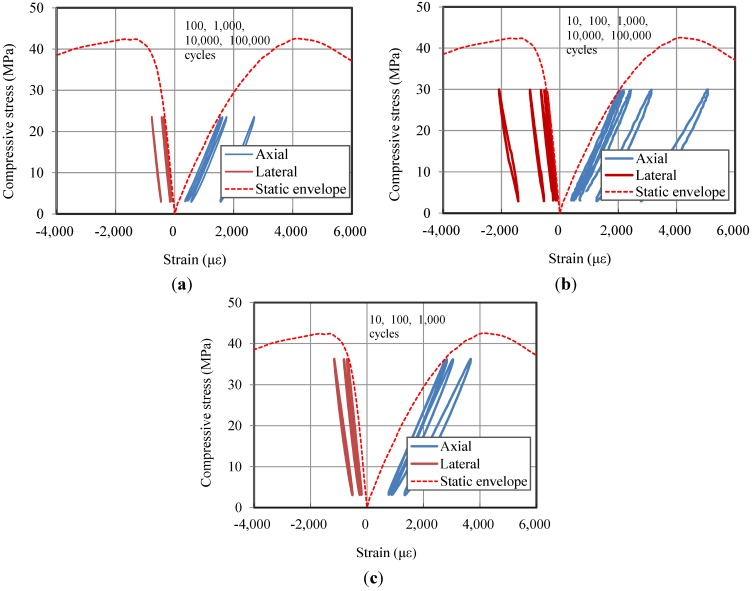
The compressive axial and lateral behavior of HPFRCC under repeated loading conditions:(**a**) the result of the 55% specimen; (**b**) the result of the 70% specimen; and (**c**) the result of 85% specimen.

Figure 7 shows the compressive behavior under repeated loads together with the static envelope. The figure indicates that the stiffness of the specimen is reduced with an increase in the number of load cycles. In addition, the residual axial strain and lateral strain both increase with the number of load cycles. When a higher level of stress is applied, the residuals increase relatively faster.

### 3.3. Damage Induced

Damage induced under repeated loads can be estimated by indirect measures, such as residual axial strain, residual lateral strain and specific crack area.

**Figure 8:**
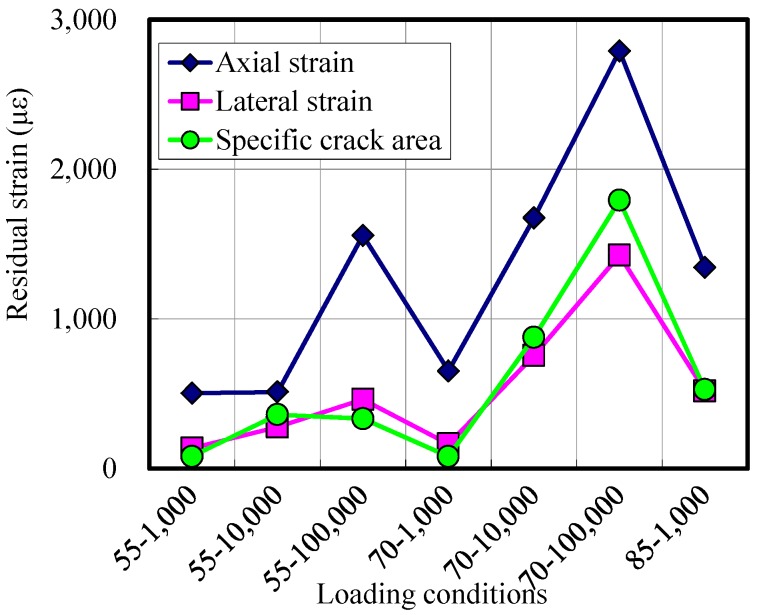
Residual strain and specific crack areas after the loads are applied.

**Figure 9:**
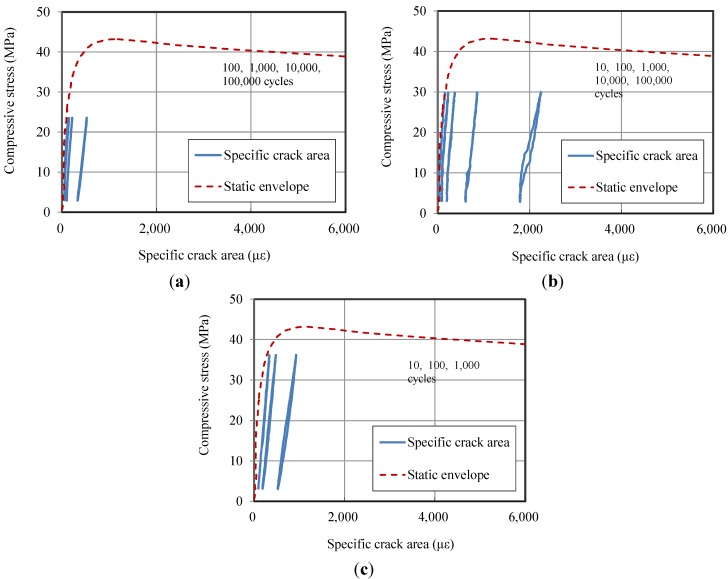
The evolution of damage to HPFRCC in repeated loading conditions:(**a**) the maximum stress is 55% of the static strength; (**b**) the maximum stress is 70% of the static strength; and (**c**) the maximum stress is 85% of the static strength.

Figure 8 shows these residual values after loads are applied. Residuals are measured at the final cycle when the applied load reaches a minimum value. It is observed overall that the residuals increase when the applied load increases or when the number of load cycles increases. Figure 9 shows the residual specific crack areas with the static damage envelope. The figure indicates that the residual crack area increases faster as the applied load gets higher.

From the observations from Figure 8 and Figure 9, it can be noticed that when the applied maximum stress is 55% of the static strength, the residual axial strain increases only 3-times after 100,000 cycles. Moreover, the residual lateral strain and the residual specific crack area are below 500 με, even after 100,000 cycles of loads. From this, it can be interpreted that a 55% compressive stress level of static strength does not induce severe damage inside the specimen. This trend coincides with the findings of other studies [20,22].

When the applied maximum stress is 70% of the static strength, the damage increased rapidly. The residual axial strain, lateral strain and specific crack area increase in proportion to the number of load cycles. For the specimen for which the applied maximum stress is 85% of the static strength, the specific crack area increases up to 1500 με, even after 1000 cyclic loads.

### 3.4. Chloride Permeability

**Figure 10:**
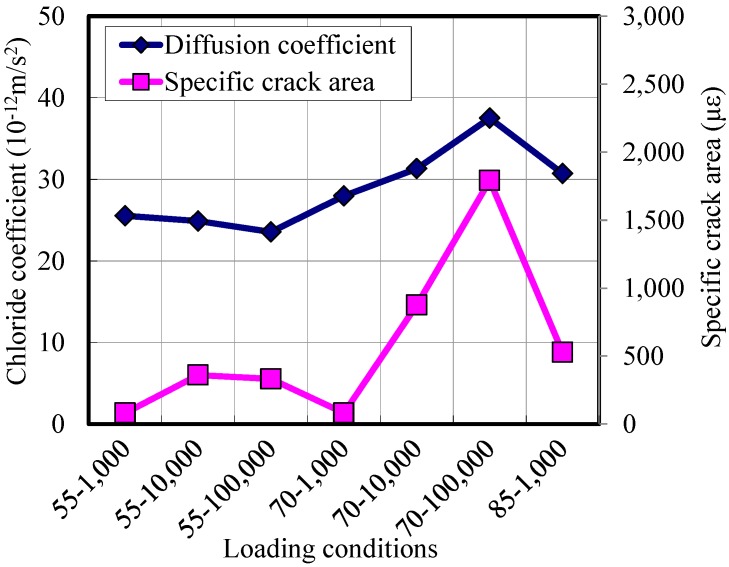
Chloride diffusion coefficients of damaged HPFRCC specimens.

The chloride permeability is measured with the specimens where damage had been induced by the repeated compressive loads. Figure 10 shows the measured diffusion coefficients together with residual specific crack areas. Overall, the chloride coefficients increase when the residual crack area increases. That is to say, as the specimen was loaded with a higher stress level or a larger number of loads, the crack area increases, resulting in an increase of diffusion coefficients.

For the specimens where the maximum applied stress was 55%, the diffusion coefficients did not change much, even when the number of applied loads increased from 1000 to 100,000. This trend coincides with the findings of Samaha and Hover [23] that microcracks in concrete at stress levels below 75% of the compressive strength did not affect the mass transport properties of the concrete.

When the maximum applied stress was 70% of the strength, the diffusion coefficients increased with an increase in the load cycles. However, the diffusion coefficients increased only 50%, even though the residual crack area increases up to 6-times, compared to the less damaged specimens (55–1000). This is due to the fact that the microcracks become unstable and begin to propagate at stresses between 70% and 90% of the compressive strength [22]. Therefore, the crack does not coalesce, and the size of the crack is kept small enough to not significantly increase the diffusion coefficients.

### 3.5. Comparison with Plain Concrete Specimens

**Figure 11:**
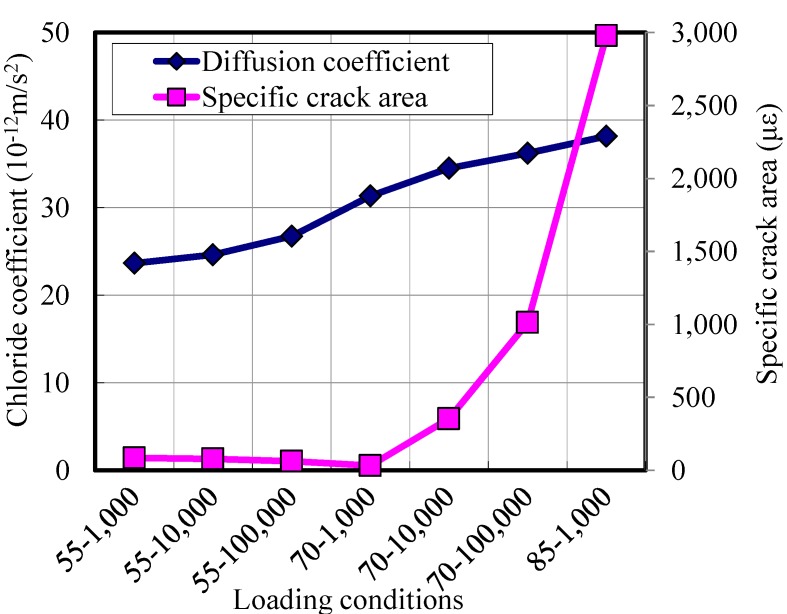
Chloride diffusion coefficients of damaged plain concrete specimens.

In order to enhance the understanding of the outcome, the results of the tests of HPFRCC are compared with those of plain concrete. Figure 11 shows the chloride diffusion coefficients of plain concrete specimens with residual specific crack areas. The figure shows that the amount of damage induced by repeated loads is related with the stress level and number of cycles, partially. When the stress level is 70%, the damage increases with an increase in the number of cycles. However, when the stress level is 55%, the damage decreases a little with the number cycles. This decrease can be observed in Banthia and Bhargava’s work [14] in which a reduction in the permeability for both plain and FRC was observed in the early stage of loads.

Figure 11 also shows that the amount of damage induced by repeated loads is related with the diffusion coefficient. As the damage increases, the diffusion coefficient increases accordingly. However, the diffusion coefficient increases only 60%, even though the residual crack area increases more than 6-times. These observations are similar to the behavior of HPFRCC.

**Figure 12:**
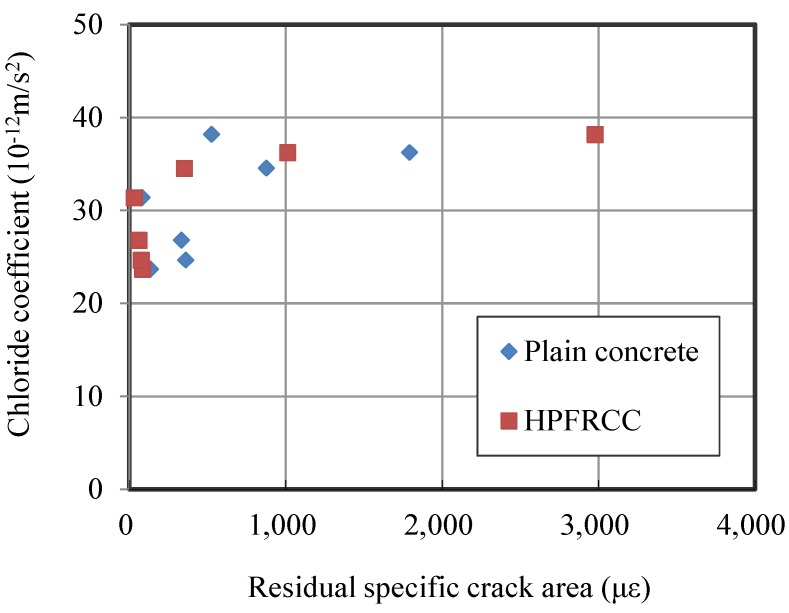
Chloride diffusion coefficients with respect to the residual specific crack area.

The diffusion coefficients of damaged HPFRCC and plain concrete are shown in Figure 12 with respect to the damage induced. The figure indicates that the diffusion coefficients are mainly correlated with the crack areas. However, the type of materials, *i.e.*, whether specimens were made of HPFRCC or plain concrete, does not influence the chloride coefficients in this experiment.

The review of the literature confirms that fine cracks distribute evenly when a large deformation is applied on the HPFRCC specimens [5,6,8,18]. For example, under a flexural or direct tensile load, the crack width of loaded HPFRCC is kept below 100 μm before the localization of cracks that typically occurs when the tensile strain reaches 2%. Therefore, the transport properties of HPFRCC are significantly improved (lower than those of plain concrete or mortar) due to its tight crack width control even though the large deformation is applied to the HPFRCC [11,12].

When compressive loads are applied on the specimens, the cracks induced in the specimens are not widened as are in the tensile loading conditions. The cracks are supposed to be very small and narrow, and these microcracks are stable below 70% and 90% of the compressive strength [22]. Therefore, it is known that compressive loads have little effect on the permeability of concrete [16,23,24].

Clearly, because permeability is affected by crack width in the concrete, bridging the microcracks before they are localized is far more beneficial to restricting fluid transport [13,14]. However, when the load is applied compressively, the deformation or strain of specimens is kept small compared to that of flexural or tensile specimens. Thus, it is considered that no significant enhancement in chloride permeability is achieved for the HPFRCC specimens in compressive loads.

Because one type of plain concrete is compared in Figure 12, but the type (or strength) of concrete influences the chloride coefficient, the chloride coefficients of plain concrete can increase or decrease with respect to the concrete properties [25]. However, since the diffusion coefficient is closely related to the crack width and not dependent on material parameters [16], it can be concluded that the use of HPFRCC in a compressive loading condition up to 85% of the maximum strength cannot give a significant reduction in the diffusion coefficient, compared to ordinary concrete.

## 4. Conclusions

The present study explores the mechanical properties of HPFRCC under static and repeated loading conditions and the chloride permeability of damaged specimens:

(1) Static mechanical tests show that the material used in this study has the typical characteristics of ductile HPFRCC. In the flexural test, specimens sustained the load continuously, even after peak load, and strain hardening behavior was observed, with finely-distributed microcracks.
(2) Artificial damage was induced by applying repeated loads on the specimen. The results of HPFRCC specimen tests show that the residual axial strain, lateral strain and specific crack area increase due to the damage induced by repeated loads. However, the chloride diffusion coefficient does not increase significantly. The chloride diffusion coefficient increases up to 1.5-times, whereas the specific crack area increases up to 3-times with an increase of damage. Plain concrete specimens also show the same trends as the HPFRCC specimens. This is due to the fact that the repeated compressive loads applied in this test induce only microcracks that cannot cause any dramatic increase of permeability, regardless of the material type.
(3) When a large deformation, causing a large amount of cracks, is applied to the specimen, finely-distributed cracks of HPFRCC can be a benefit in terms of the restriction of the crack width and reducing chloride permeability. However, for a moderate compressive load, which was below 85% of the static strength, the benefit of HPFRCC on the chloride diffusion coefficient is not significant. Because the compressive load does not induce dominant wide cracks in a stress level below 85% of the static strength, only small-scale damage is induced, even for plain concrete. Therefore, HPFRCC and plain concrete do not show significant differences in crack areas and chloride diffusion coefficients.
